# A systematic review of microbiological applications in drowning and near-drowning 

**Published:** 2022-01

**Authors:** Seyed Shahram Mirzamani

**Affiliations:** ^*a*^ DVM-MPH, PhD, Candidate in Microbiology, Deputy of Health, Department of Health, Iranian Strategic Naval Force; Student of Department of Biology, School of Basic Sciences, Islamic Azad University, Science and Research Branch, Tehran, Iran.

## Abstract

**Background:**

Drowning and near-drowning are the consequences of recreational activities and natural disasters. The purpose of this study was to review the microbiology literature on drowning and near-drowning and to understand its applications in the diagnosis and treatment of these injuries and diseases.

**Methods:**

This study is the result of a short review on publications extracted by searching in scientific databases including Google Scholar, PubMed and Semantic Scholar in the period from 1961 to 2021, using the keywords "Microbiology" and "Drowning" or "Near-Drowning".

**Results:**

A search in scientific databases revealed 184 articles related to "drowning" and "microbiology" and 84 articles related to "near-drowning" and "microbiology", of which 65 and 28 articles have been published in the last twenty years, respectively. Of these, 43 articles were used for this study. Regarding the microbiology of drowning and near-drowning, no study was conducted in the Islamic Republic of Iran in this field. The findings of the present study showed that:

• Near-drowning in its victims causes physical and mental injuries, and invasive polymicrobial and fungal pneumonia, brain abscesses, and sepsis.

• They are mainly associated with numerous bacterial agents e.g. Aeromonas spp., Nocardia spp., Vibrio spp., Photobacterium spp., Burkholderiapseudomallei, Pseudomonas spp., Plesiomonas shigelloides, and Shewanella spp. and Fungal and Protozoan pathogens are limited to Aspergillus spp., Scedosporium apiospermum, and Rhizopus spp., and Cryptosporidium parvum (rare), respectively.

• If bacterial, fungal, and protozoa infections are not properly diagnosed and treated in drowning victims, they can lead to death in intensive care units and even long after discharge from the hospital.

• Deaths from drowning and near-drowning are candidates for organ transplants, especially lung, bone, liver, and heart.

• Clinical, environmental, forensic, and eco-microbio-epidemilogy studies in pre-and post-mortem drowning victims are being developed using cultured-based and molecular methods such as PCR, LAMP, NGS, and Metagenomics, etc. to determine the drowning microbiome pattern.

**Conclusions:**

Several cases of infections and diseases caused by drowning and near-drowning have been reported among the victims of recreational activities and natural disasters in different countries. Therefore, eco-microbio-epidemiological study of drownings in our country, where there is an average of 1,200 annual drownings on the north and south coasts and inland waters is essential. Even predicting tsunami and destructive storms on the shores of the Caspian Sea and the Oman Sea are very important in determining the microbiome pattern of natural aquatic areas and the role of microbes in diseases and complications caused by drowning. Finally, a national protocol should be developed for microbial monitoring of drowning victims from the scene of water accidents to the hospital bed and on the autopsy table.

**Keywords:**

Microbiology, Microbiome, Drowning, Near-Drowning, Natural Disasters

